# OP46 Cost Effectiveness Of Active Middle Ear Implant For Individuals With Untreated Sensorineural Hearing Loss In Brazil

**DOI:** 10.1017/S0266462325101049

**Published:** 2025-12-29

**Authors:** Erica Aranha Suzumura, Bettina Schlick, Christina Schwarz, Thomas Dejaco, Michael Urban

## Abstract

**Introduction:**

Individuals with moderate to severe sensorineural hearing loss (SNHL), who are unable to use conventional hearing aids (CHA) for medical reasons, have no publicly financed treatment options in Brazil. This study aimed to evaluate the cost effectiveness of active middle ear implants (aMEI) for Brazilian adults with this health condition.

**Methods:**

A Markov model with cohort simulation over lifetime was developed. To populate the model, a systematic review was conducted to identify implant-related complications and utilities. Cost of aMEI was based on the price proposed for public reimbursement. Pre-, peri-, and postoperative costs were obtained from the Brazilian public healthcare system list of procedures (SIGTAP). Costs of untreated SNHL in Brazilian adults were obtained from the World Health Organization report (2015) and adjusted for 2024 BRL using purchasing power parities (PPP) for gross domestic product (GDP). Probabilistic sensitivity analyses (PSA) were performed to evaluate the robustness of the results. Cost results were presented in 2024 BRL and USD (conversion using PPP for GDP).

**Results:**

The base case result showed that, over a lifetime, the expected cost of aMEI treatment was BRL62,177 (USD26,951), compared with BRL5,806 (USD2,517) in an untreated adult. The expected quality-adjusted life years (QALYs) for a patient treated with aMEI was 10.53, compared with 9.03 in a patient left untreated. The incremental cost-effectiveness ratio was BRL37,699 (USD16,341) per QALY gained, which is cost effective considering the current willingness-to-pay threshold adopted in Brazil of one GDP per capita (BRL55,400 [USD24,014] in 2024). PSA showed that results of the intervention were robust and cost effective in most of the simulations.

**Conclusions:**

aMEI is a cost-effective hearing solution for adults with moderate to severe SNHL in Brazil considering the perspective of the healthcare system. Assuring the access of aMEI to adults with moderate to severe SNHL who are unable to use CHA for medical reasons is important to promote health equity in Brazil.

